# Association Between Change in Prognostic Nutritional Index During Neoadjuvant Therapy and Dental Occlusal Support in Patients with Esophageal Cancer Under Neoadjuvant Therapy: A Retrospective Longitudinal Pilot Study

**DOI:** 10.3390/nu16244383

**Published:** 2024-12-19

**Authors:** Reiko Yamanaka-Kohno, Yasuhiro Shirakawa, Mami Inoue-Minakuchi, Aya Yokoi, Kazuhiro Noma, Shunsuke Tanabe, Naoaki Maeda, Toshiyoshi Fujiwara, Manabu Morita, Daisuke Ekuni

**Affiliations:** 1 Department of Preventive Dentistry, Division of Dentistry, Okayama University Hospital, Okayama 700-8558, Japan; 2 Department of Gastroenterological Surgery, Okayama University Graduate School of Medicine, Dentistry and Pharmaceutical Sciences, Okayama 700-8558, Japan; yasuwr@city-hosp.naka.hiroshima.jp (Y.S.); knoma@md.okayama-u.ac.jp (K.N.); stanabe114@cc.okayama-u.ac.jp (S.T.); p41n53io@s.okayama-u.ac.jp (N.M.); toshi_f@md.okayama-u.ac.jp (T.F.); 3 Department of Surgery, Hiroshima City Hiroshima Citizens Hospital, Hiroshima 730-8518, Japan; 4 Department of Oral Rehabilitation and Regenerative Medicine, Faculty of Medicine, Dentistry and Pharmaceutical Sciences, Okayama University, Okayama 700-8558, Japan; minakuchimami@gmail.com; 5 Department of Preventive Dentistry, Faculty of Medicine, Dentistry and Pharmaceutical Sciences, Okayama University, Okayama 700-8558, Japan; yokoi-a1@cc.okayama-u.ac.jp (A.Y.); m.morita@tumh.ac.jp (M.M.); dekuni7@md.okayama-u.ac.jp (D.E.); 6 Department of Oral Health Sciences, Takarazuka University of Medical and Health Care, 1, Midorigaoka, Hanayashiki, Takarazuka 666-0162, Japan

**Keywords:** esophageal cancer, prognostic factors, nutrition, neoadjuvant therapy, dental occlusion

## Abstract

**Background:** A high prognostic nutritional index (PNI) is associated with good prognosis in patients with esophageal cancer. However, nutritional status often decreases during neoadjuvant therapy. Functional tooth units (FTUs) provide an index for the status of posterior occlusal support. We have previously reported that low PNI is related to low FTUs. **Objectives:** The purpose of this study was to retrospectively examine whether the status of occlusal support relates to changes in PNI during neoadjuvant therapy in patients with esophageal cancer. **Methods:** This study included 34 patients who underwent neoadjuvant therapy before esophagectomy (32 men, 2 women; age, 36–82 years) in 2012 at Okayama University Hospital. Patients were divided into the good occlusal support group (FTUs ≥ 11, *n* = 18) or poor occlusal support group (FTUs < 11, *n* = 16), and changes in PNI during neoadjuvant therapy were investigated. **Results:** PNI decreased significantly after neoadjuvant therapy, particularly in the good occlusal support group, and became more dispersed after neoadjuvant therapy. Decreases in PNI after neoadjuvant therapy showed a significant positive correlation with good occlusal support by multiple regression analysis (*p* = 0.03). The proportions of patients provided with nutritional intervention (*p* = 0.02) or early dental intervention (*p* = 0.04) were lower in the good occlusal support group than in the poor occlusal support group. **Conclusions:** Even in patients with esophageal cancer with good occlusal support experienced significant declines in PNI during neoadjuvant therapy, potentially due to delayed nutritional and dental interventions. Early multidisciplinary interventions are thus recommended for all patients, regardless of preoperative dental or nutritional status.

## 1. Introduction

For advanced esophageal cancer, neoadjuvant therapy has shown a 2-year survival benefit of 5.1% compared with esophagectomy alone [1,2]. While evidence suggests that neoadjuvant therapy can offer a survival advantage, associated toxicities can exacerbate poor nutritional status [3]. In addition, patients with esophageal cancer are often malnourished, and optimizing their nutrition is difficult [1]. A review demonstrated the uncertainty surrounding the optimal nutritional approach for patients with resectable esophageal cancer undergoing neoadjuvant therapy prior to esophagectomy [3]. No consensus has been reached regarding a standard of care for the optimal nutritional approach [4]. Elucidating factors related to changes in nutritional status during neoadjuvant therapy could help maintain adequate nutritional status during neoadjuvant therapy.

Prognostic scores based on nutritional status have been given increasing attention in patients with esophageal cancer [5,6], and these indices might be useful to control nutritional status during neoadjuvant chemotherapy. Both the prognostic nutritional index (PNI) described by Onodera et al. and controlling nutritional status (CONUT) score are simple to calculate from peripheral blood data, easy to interpret, and highly accurate for predicting the prognosis of patients with esophageal cancer [5,7]. In these patients, CONUT score might predict prognosis more accurately than PNI at 14 days after surgery [8]. On the other hand, PNI is more convenient and useful before surgery, including during preoperative treatment [8]. PNI would therefore be appropriate for evaluating nutritional status during neoadjuvant therapy. As toxicities associated with neoadjuvant therapy can exacerbate poor nutritional status [3], PNI might decrease during neoadjuvant therapy in patients with esophageal cancer.

Patients with esophageal cancer who underwent esophagectomy with a low PNI showed fewer functional tooth units (FTUs) compared to those with a high PNI [9]. FTUs reflect the status of posterior occlusal support and are associated with nutritional status and masticatory ability [10,11,12]. FTUs show a potential cause-and-effect pathway with masticatory function and vegetable intake in adults with advanced periodontitis [13]. In addition, the low number of FTUs in patients with preoperative gastric cancer was a risk factor for a greater percentage of body weight loss at 1 and 3 months postoperatively [14].

Given this background, we hypothesized that PNI might decrease during neoadjuvant therapy, and that poor occlusal support might be related to a greater decrease in PNI during neoadjuvant therapy in patients with esophageal cancer. The purpose of this study was to retrospectively confirm whether PNI decreases during neoadjuvant therapy, and to retrospectively examine the association between occlusal support and changes in PNI during neoadjuvant therapy for patients with esophageal cancer.

## 2. Materials and Methods

### 2.1. Human Rights Statement

This study was approved by the Ethics Committee at Okayama University Graduate School of Medicine, Dentistry and Pharmaceutical Sciences and Okayama University Hospital (20/11/2017/No. Ken1711-027). All procedures followed were in accordance with the ethical standards of the responsible committee on human experimentation (institutional and national) and with the 1964 Declaration of Helsinki and later versions. All patients had the right to opt out from this retrospective study, and patient anonymity was preserved.

### 2.2. Study Populations

This retrospective study included 34 patients (32 men, 2 women; mean age, 65.4 ± 8.9 years) who had received neoadjuvant therapy without radiation therapy, among the 73 patients with esophageal cancer who had undergone esophagectomy at Okayama University Hospital between January and December 2012.

### 2.3. General Patient Status

The characteristics of the study population were obtained from their electronic medical records and are summarized in Table 1. Medical and dental data were collected for all subjects on the day of the first dental examination for perioperative oral management. Age, sex, cancer stage, body mass index, smoking and drinking habits, and operative procedures were recorded. Smoking and drinking habits were dichotomized into “yes” or “no”, with “yes” including current and former smokers and drinkers [9], and “no” assigned to never-smokers and never-drinkers [9]. Operative procedures were divided into two groups, comprising thoracoscopic surgery and transhiatal esophagectomy.

Blood was collected before neoadjuvant therapy and immediately before esophagectomy to obtain the following data: total white blood cell (WBC) count, total lymphocyte count, total protein, albumin, and *C*-reactive protein (CRP).

Clinical findings during the first course of neoadjuvant therapy, regimens, severity of oral mucositis, and nutritional intervention during neoadjuvant therapy were recorded from the medical chart.

Oral mucositis was diagnosed using Common Terminology Criteria for Adverse Events (CTCAE) version 5.0, as follows: Grade 1, asymptomatic or mild oral symptoms, intervention not indicated; Grade 2, moderate pain or ulcer that does not interfere with oral intake, diet modification indicated; Grade 3, severe pain interfering with oral intake; Grade 4, life-threatening consequences, urgent intervention indicated; and Grade 5, death.

Nutritional intervention was recorded in the first and second courses of neoadjuvant therapy as absence, nutritional supplement, enteral nutrition, parenteral nutrition, or unclear.

### 2.4. Dental Intervention and Status

Dental interventions including periodontal examination, oral hygiene instructions, scaling and root planning, instruction, and medication (mouthwash and ointment) were employed to prevent the onset or aggravation of oral mucositis. The timing of dental intervention was investigated and categorized into five groups: 1, before the first course of neoadjuvant therapy; 2, during the first course of neoadjuvant therapy; 3, after the first course of neoadjuvant therapy; 4, during the second course of neoadjuvant therapy; or 5, after the second course of neoadjuvant therapy. Oral hygiene status was evaluated by well-trained dentists at the first visit to the Hospital Dentistry Clinic, and it was scored on an ordinal scale divided into five groups (1, good; 2, slightly good; 3, normal; 4, slightly poor; or 5, poor).

The following dental status indicators were evaluated: number of teeth present, Community Periodontal Index (CPI) [15], and number of FTUs [9,10,11,15,16].

Periodontitis was evaluated using the CPI. The CPI score was determined as follows: score 0, healthy periodontal condition; score 1, gingival bleeding; score 2, dental calculus and bleeding; score 3, shallow periodontal pockets (4–5 mm); and score 4, deep periodontal pockets (≥6 mm). The maximum score for the teeth of a subject was considered the CPI score for that individual [15]. Patients with no teeth for the calculation of CPI were considered lost due to periodontitis. Periodontitis was defined as CPI code 3 or 4 and no subject teeth or loss of teeth due to periodontitis.

The number of FTUs was used to evaluate posterior occlusal support without wisdom teeth, and it is widely used for evaluating dental occlusal support [9,10,11,12,13,14,16]. The number of FTUs was defined as the number of pairs of opposing posterior natural teeth and artificial teeth that are implant-supported, fixed (bridge pontics), and removable prostheses. Teeth with dental caries showing extensive coronal destruction and missing teeth were regarded as non-functional. Two opposing premolars were defined as one FTU, and two opposing molars were defined as two FTUs. The score for FTUs therefore ranged from 0 to 12, where a score of 12 represented an individual with complete posterior occlusal support and higher scores represented better posterior occlusal support. FTUs were used as an indicator of dental occlusal support and were treated as continuous variables.

### 2.5. PNI and Change in PNI Calculation

PNI was calculated according to the method described by Onodera: PNI = 10 × albumin (g/dL) + 0.005 × total lymphocyte count (/mm^3^) in peripheral blood [7]. PNI was calculated before and after neoadjuvant therapy, and the change in PNI was calculated as PNI before neoadjuvant therapy—PNI after neoadjuvant therapy.

### 2.6. Statistical Analyses

Sample sizes were not calculated because this was a pilot study, and the subjects were all patients with esophageal cancer who were provided with neoadjuvant chemotherapy at Okayama University Hospital in 2012. The normality of data was investigated using histograms, the Shapiro–Wilk test, normal Q–Q plots, skewness, and kurtosis [17]. We confirmed the non-normal distribution of PNI before neoadjuvant therapy (*p* < 0.01) and the normal distribution of PNI after neoadjuvant therapy (*p* = 0.11) by using the Shapiro–Wilk test and the difference in PNI between before and after neoadjuvant therapy (*p* = 0.09), as well as other methods [17]. Non-parametric tests including the Wilcoxon signed-rank test and Mann–Whitney *U* test were used for statistical analyses concerning PNI before neoadjuvant therapy. The difference in PNI between before and after neoadjuvant therapy was compared using the Wilcoxon signed-rank test. Correlations between a decrease in PNI (before neoadjuvant therapy—after neoadjuvant therapy) and other parameters were analyzed using the Spearman correlation coefficient. Continuous and ordinal scales were treated as parameters, and a description (reference range or parameter) was indicated for ordinal scales. Multiple regression analysis was used for comparing correlations between a decrease in PNI and parameters. In the multiple regression analysis, all parameters were regarded as independent variables, and the parameters showing a Spearman correlation coefficient with *p* < 0.10 were added to the model.

Patients were divided into two groups of almost equal size with good or poor occlusal support. The cut-off for FTUs was 11 (the median value); individuals with ≥11 FTUs were defined as having good dental occlusal support (*n* = 18), while those with <11 FTUs were defined as having poor dental occlusal support (*n* = 16). The difference in PNI between before and after neoadjuvant therapy in the good and poor dental occlusal support groups was compared using the Wilcoxon signed-rank test. The difference in PNI between the good and poor dental occlusal support groups was compared before and after neoadjuvant therapy using the Mann–Whitney *U* test. Clinical findings during neoadjuvant therapy were compared between the good and poor dental occlusal support groups. The regimen, severity of oral mucositis, nutritional intervention with the first course of neoadjuvant therapy, timing of dental intervention, oral hygiene status, and periodontal status were parameterized into two-valued independent variables. The two-valued independent parameters for clinical findings during neoadjuvant therapy were compared between the good and poor dental occlusal support groups using the one-sided Fisher’s exact test. Results were considered significant at *p* < 0.05. All statistical analyses were performed using SPSS for Windows version 26 (SPSS Inc., Chicago, IL, USA).

## 3. Results

Table 1 shows the characteristics including blood data and dental status of the study population on the day of the first dental examination for perioperative oral management.

**Figure 1:**
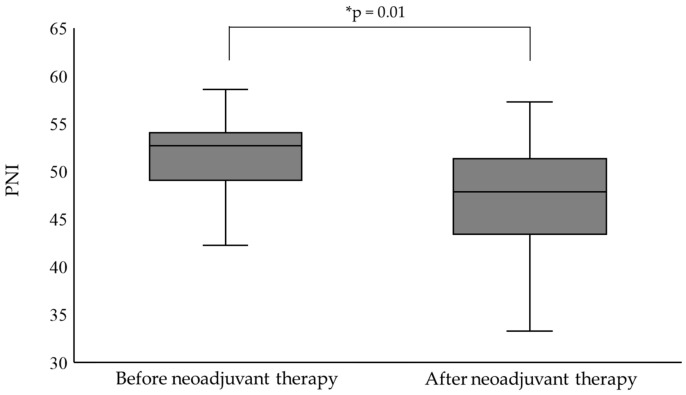
Difference in PNI before and after neoadjuvant therapy in all patients (*n* = 34). PNI was significantly lower after neoadjuvant therapy than before neoadjuvant therapy (* Wilcoxon signed-rank test). PNI, prognostic nutritional index.

PNI after neoadjuvant therapy was significantly decreased compared to before neoadjuvant therapy (Figure 1).

Table 2 shows the correlation between the decrease in PNI during neoadjuvant therapy and each parameter. In multiple regression analysis, good occlusal support displayed a significant positive correlation with the decrease in PNI during neoadjuvant therapy, after adjusting for age (Table 3).

**Figure 2:**
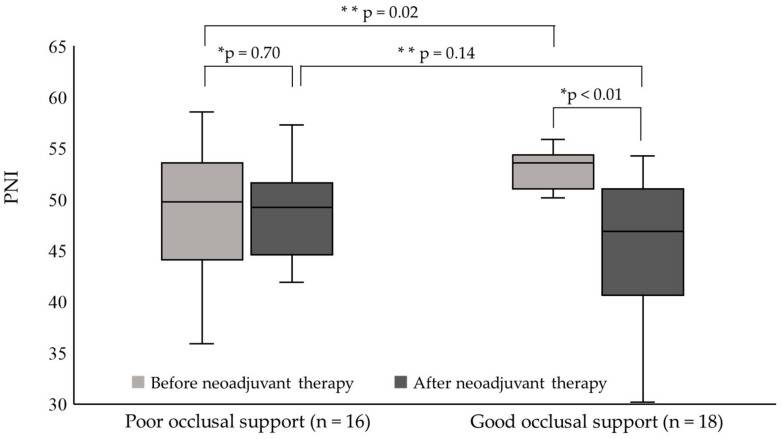
Difference in PNI between before and after neoadjuvant therapy in the good and poor occlusal support groups. In the good occlusal support group, PNI was significantly lower after neoadjuvant therapy than before neoadjuvant therapy (* Wilcoxon signed-rank test). Before neoadjuvant therapy, PNI was significantly higher in the good occlusal support group than in the poor occlusal support group (** Mann–Whitney *U* test). After neoadjuvant therapy, no significant difference in PNI was seen between the good and poor occlusal support groups (** Mann–Whitney *U* test).

Figure 2 shows PNI before and after neoadjuvant therapy in the good and poor occlusal support groups. In the good occlusal support group, PNI after neoadjuvant therapy was significantly decreased compared to before neoadjuvant therapy (*p* < 0.01), and the interquartile range (IQR) widened. On the other hand, the poor occlusal support group showed no significant difference in PNI between before and after neoadjuvant therapy (*p* = 0.70), and the IQR narrowed. Table 4 shows the clinical findings during neoadjuvant therapy. The proportion of patients provided with nutritional intervention during the first course of neoadjuvant therapy was significantly lower in the good occlusal support group than in the poor occlusal support group (*p* = 0.02). While there was not significant difference the time of dental intervention (5 groups) and oral hygiene status (5 groups) in the good occlusal support group and in the poor occlusal support group, the proportion provided dental intervention before the initiation of second course of neoadjuvant therapy (2 groups) and with not good oral hygiene status (2 groups) was significantly lower in the good occlusal support group than in the poor occlusal support group (*p* = 0.04).

## 4. Discussion

In this study, we confirmed that PNI decreased significantly after neoadjuvant therapy compared to before neoadjuvant therapy. In particular, PNI in the good occlusal support group decreased significantly and showed increased variability after neoadjuvant therapy. On the other hand, the decrease in PNI in the poor occlusal support group was not significant, and variability decreased after neoadjuvant therapy. These results were completely counter to what we had expected.

The most surprising finding of this study was that good occlusal support showed a positive relationship with the decrease in PNI during neoadjuvant therapy. According to our previous study, poor occlusal support correlated with low PNI in patients with esophageal cancer who underwent esophagectomy [9]. We therefore anticipated that PNI would decrease in patients with poor occlusal support during neoadjuvant therapy. The reason for the unexpected results may be as follows. Subjects in this study were provided with neoadjuvant therapy in 2012, when the perioperative team approach started to systematically include dental intervention after neoadjuvant therapy, not before neoadjuvant therapy. Patients with poor occlusal support had received more nutritional intervention during the first course of neoadjuvant therapy because they were considered malnourished and at high risk. In fact, median PNI in the poor occlusal support group was not significantly decreased after neoadjuvant therapy, and the IQR for PNI after neoadjuvant therapy was narrowed compared to before neoadjuvant therapy. That is, nutritional intervention during neoadjuvant therapy proved quite successful for poorly nourished patients with poor occlusal support. On the other hand, PNI in patients with good occlusal support would have suggested these patients were well nourished before neoadjuvant therapy, so application of the multidisciplinary team approach might have been delayed. This suggests that all patients who undergo neoadjuvant therapy should be approached using an early multidisciplinary team including dentistry before neoadjuvant therapy, independent of patient status.

Malnutrition in patients with cancer negatively impacts clinical outcomes and increases the mortality risk [18]. Indeed, malnutrition is often a direct consequence of the treatments themselves, as one of the possible side effects [19]. Malnutrition during oncological systemic treatments, including neoadjuvant chemotherapy, can lead to a dangerous, vicious cycle [19]. Esophageal cancer, in particular, is a debilitating disease with poor prognosis, and weight loss due to malnutrition is common among patients [20]. Nutritional management is critically important for patients with esophageal cancer, and every possible countermeasure is required.

The European Society for Clinical Nutrition and Metabolism has provided practical guidelines to help healthcare providers offer optimal nutritional care for patients with cancer [21]. Despite the recognized need for adequate nutritional support in patients with cancer, nutrition is still often not prioritized in daily clinical practice [19]. In our previous study, the rate of adverse events (particularly severe oral complications) was significantly reduced during chemotherapy under a multidisciplinary team approach that included dental intervention. In addition, weight loss from chemotherapy to surgery was significantly reduced in the group that received care before neoadjuvant chemotherapy [22]. This highlights the importance of maintaining oral health to ensure continued oral intake during neoadjuvant therapy, particularly for patients with good occlusal support who had been considered to not need nutritional support.

In a multicenter analysis, perioperative oral management exerted significant positive effects on perioperative serum albumin levels in patients who underwent surgery under general anesthesia [23]. That study noted that perioperative oral management including oral care, removal of chronic dental infections, and prosthodontic treatments exerted positive effects on perioperative serum albumin levels by inhibiting early postoperative decreases in serum albumin levels [23]. Similar positive effects of oral management on nutritional status might be seen during neoadjuvant therapy in patients with esophageal cancer. We have sometimes encountered cases in which oral management effectively prevented the worsening of and infection in oral mucositis [24]. More studies are required to clarify associations between oral management and nutritional status during neoadjuvant therapy in patients with esophageal cancer.

In terms of the primary outcome of decreases in PNI during neoadjuvant therapy, sample size was considered in this pilot study. The subjects were all patients with esophageal cancer (*n* = 34) who were provided with neoadjuvant chemotherapy at Okayama University Hospital in 2012. Given an effect size of 0.47, and two independent variables, a significance level (α) of 0.05, and statistical power of 0.8, G*Power version 3.1.9.7 (a tool to compute statistical power analyses) calculated that 24 samples would be required [25,26]. The sample size of 34 in this study was thus considered sufficient.

This study has some limitations. First, we performed many statistical analyses in this study. For example, Bonferroni correction should have been performed for the Wilcoxon signed-rank test and Mann–Whitney *U* test because these were performed two times, respectively (Figure 2). The significance level was *p* = 0.025 (0.05/2) according to Bonferroni correction. There were significant differences between PNI before neoadjuvant therapy in the poor and good occlusal groups, and also between PNI before and after neoadjuvant therapy in the good occlusal support group (Figure 2). Furthermore, as shown in Table 4, Bonferroni correction should also be performed for Fisher’s exact test because this test was performed six times. In this case, the significance level was *p* = 0.083 (0.05/6), indicating no significant differences (Table 4). Even in the absence of significant differences for the data shown in Table 4, the clinical findings presented in Table 4 did not deal with primary outcomes, but only supplementary subgroup analyses, and the findings suggested interesting directions for future studies and for potential avenues toward resolving clinical problems. Second, all subjects were collected from only a single hospital, the sample size was small, and the data were relatively old. Third, this study did not reveal whether the effects of early dental intervention improved nutritional status, especially for patients with esophageal cancer with good occlusal support. However, this study provided useful and suggestive information for maintaining nutritional status during neoadjuvant therapy in patients with esophageal cancer. An early multidisciplinary team approach including dentistry seems likely to be more effective, even for well-nourished patients with good occlusal support. Prospective, multicenter, observational cohort studies are now needed to determine the optimal strategy for maintaining adequate nutritional status during neoadjuvant therapy for patients with esophageal cancer.

## 5. Conclusions

Decreases in PNI during neoadjuvant therapy correlated significantly with good occlusal support in patients with esophageal cancer. Patients with good occlusal support were well nourished before neoadjuvant therapy and so were not provided with early nutritional intervention and dental interventions, unlike patients with poor occlusal support. A system capable of providing an early perioperative multidisciplinary team approach to everyone would be ideal, independent of nutritional and oral status before neoadjuvant therapy.

## Figures and Tables

**Table 1:** Characteristics of the study population (*n* = 34).

Parameter	Value
Age (years)	66.0 (60.8–72.0)
Sex (male)	32 (94.1)/2 (5.9)
Cancer stage (0–II/III, IV)	12 (35.3)/22 (64.7)
Body mass index (kg/m^2^)	22.9 (18.7–25.2)
Smoking habit (yes/no)	31 (91.2)/3 (8.8)
Drinking habit (yes/no)	31 (91.2)/3 (8.8)
Operative procedures	
Thoracoscopic surgery/Transhiatal esophagectomy	26 (76.5)/8 (23.5)
WBC, ×10^3^/μL	
Before neoadjuvant therapy	6.93 (5.86–8.44)
After neoadjuvant therapy	5.41 (4.62–7.33)
Total lymphocyte count, ×10^3^/μL	
Before neoadjuvant therapy	1.89 (1.41–2.21)
After neoadjuvant therapy	1.64 (1.29–2.08)
Total protein, g/dL	
Before neoadjuvant therapy	7.20 (6.90–7.40)
After neoadjuvant therapy	6.65 (6.18–7.00)
Albumin, g/dL	
Before neoadjuvant therapy	4.20 (4.00–4.43)
After neoadjuvant therapy	3.90 (3.58–4.23)
CRP, mg/dL	
Before neoadjuvant therapy	0.11 (0.05–0.91)
After neoadjuvant therapy	0.10 (0.50–1.09)
Regimen in first course of neoadjuvant therapy	
Docetaxel, cisplatin and 5-fluorouracil	10 (29.4)
5-Fluorouracil and cisplatin	19 (55.9)
Nedaplatin	1 (2.9)
Nedaplatin + 5-fluorouracil	1 (2.9)
Nedaplatin + 5-fluorouracil and cisplatin	1 (2.9)
Cisplatin + irinotecan	1 (2.9)
Docetaxel + tegafur, gimeracil, and oteracil	1 (2.9)
Nothing	0 (0.0)
Unclear (performed at another hospital)	0 (0.0)
Regimen in second course of neoadjuvant therapy	
Docetaxel, cisplatin, and 5-fluorouracil	9 (26.5)
5-fluorouracil and cisplatin	12 (35.3)
Nedaplatin	3 (8.8)
Nedaplatin + 5-fluorouracil	0 (0.0)
Nedaplatin + 5-fluorouracil, and cisplatin	1 (2.9)
Cisplatin + irinotecan	0 (0.0)
Docetaxel + tegafur, gimeracil, and oteracil	1 (2.9)
Irinotecan	1 (2.9)
Nothing	6 (17.6)
Unclear (performed at another hospital)	1 (2.9)
Oral mucositis	
In first course of neoadjuvant therapy (CTCAE ver. 5)	
Grade 0	0 (0)
Grade 1	21 (61.8)
Grade 2	7 (20.6)
Grade 3	6 (17.6)
Grade 4	0 (0.0)
In second course of neoadjuvant therapy (CTCAE ver. 5)	
Grade 0	0 (0.0)
Grade 1	18 (52.9)
Grade 2	5 (14.7)
Grade 3	4 (11.8)
Grade 4	0 (0.0)
Not applicable	7 (20.6)
Nutritional interventions	
In first course of neoadjuvant therapy	
Absence	20 (58.8)
Nutritional supplement	0 (0.0)
Enteral nutrition	2 (5.8)
Parenteral nutrition	12 (35.3)
Unclear	0 (0.0)
In second course of neoadjuvant therapy	
Absence	23 (67.6)
Nutritional supplement	1 (2.9)
Enteral nutrition	1 (2.9)
Parenteral nutrition	8 (23.5)
Unclear (performed in another hospital)	1 (2.9)
Timing of first dental examination	
1: Before first course of neoadjuvant therapy	6 (17.6)
2: During first course of neoadjuvant therapy	9 (26.5)
3: After first course of neoadjuvant therapy	2 (5.9)
4: During second course of neoadjuvant therapy	4 (11.8)
5: After second course of neoadjuvant therapy	13 (38.2)
Oral hygiene status at first dental examination	
1: Good	7 (20.6)
2: Slightly good	8 (23.5)
3: Normal	3 (8.8)
4: Slightly poor	10 (29.4)
5: Poor	6 (17.6)
Number of teeth present	22.0 (13.0–27.0)
Periodontal status	
Healthy periodontal condition	2 (0.0)
Gingival bleeding	4 (11.8)
Dental calculus and bleeding	2 (5.8)
Shallow periodontal pockets (4–5 mm)	9 (26.5)
Deep periodontal pockets (≥6 mm)	15 (44.1)
No subject teeth	2 (5.8)
Dental occlusal support (FTUs)	11.0 (6.0–12.0)

Values are given as median (interquartile range) or number (percentage). WBC, white blood cell count; CRP, *C*-reactive protein; FTUs, functional tooth units.

**Table 2:** Association between changes in PNI between before chemotherapy and after neoadjuvant therapy, and related parameters (*n* = 34).

Parameter	Description (Reference Range or Parameter)	Spearman Correlation Coefficient	*p* Value
Age	Continuous	0.32	0.07
Body mass index (kg/m^2^)	Continuous	−0.16	0.36
WBC before neoadjuvant therapy	Continuous	0.002	0.99
Total protein before neoadjuvant therapy	Continuous	0.09	0.61
CRP before neoadjuvant therapy	Continuous	−0.20	0.26
Timing of first dental intervention	Ordinal (1: early–5: delay)	0.22	0.21
Oral hygiene status at first dental examination	Ordinal (1: good–5: poor)	−0.12	0.52
Number of present teeth	Continuous	0.05	0.77
Dental occlusal support	Continuous (1–12 FTUs)	0.52	<0.01

WBC, white blood cell count; CRP, *C*-reactive protein.

**Table 3:** Correlations between decrease in PNI and parameters on multiple regression analysis (*n* = 34).

Parameter	Description	Standardized Coefficient	Confidence Interval	*p* Value
Age	Continuous	0.29	−0.02–0.51	0.06
Dental occlusal support	Continuous	0.47	0.29–1.38	<0.01

**Table 4:** Clinical findings during first course of neoadjuvant therapy (*n* = 34).

Parameter	Poor Occlusal Support Group (*n* = 16)	Good Occlusal Support Group (*n* = 18)	*p* Value *
Regimen (%)			
Docetaxel, cisplatin, and 5-fluorouracil	3 (18.8)	7 (38.9)	0.18
Others	13 (81.3)	11 (61.1)	
Oral mucositis (CTCAE ver. 5)			
Grade 1	9 (56.3)	12 (66.7)	0.39
Grade 2 or 3	7 (43.8)	6 (33.3)	
Nutritional intervention in first course of neoadjuvant therapy			
Absence	6 (37.5)	14 (77.8)	0.02
Presence	10 (62.5)	4 (22.2)	
Time of dental intervention (5 groups)			
1: Before first course of neoadjuvant therapy	3 (18.8)	3 (16.7)	0.20
2: During first course of neoadjuvant therapy	7 (43.8)	2 (11.1)	
3: After first course of neoadjuvant therapy	1 (6.3)	1 (5.6)	
4: During second course of neoadjuvant therapy	1 (6.3)	3 (16.7)	
5: After second course of neoadjuvant therapy	4 (25.0)	9 (50.0)	
Time of dental intervention (2 groups)			
Before start of second course of neoadjuvant therapy	11 (68.8)	6 (33.3)	0.04
After start of second course of neoadjuvant therapy	5 (31.3)	12 (66.7)	
Oral hygiene status (5 groups)			
1: Good	0 (0.0)	7 (38.9)	0.05
2: Slightly good	5 (31.3)	3 (16.7)	
3: Normal	2 (12.5)	1 (5.6)	
4: Slightly poor	5 (31.3)	5 (27.8)	
5: Poor	4 (25.0)	2 (11.1)	
Oral hygiene status (2 groups)			
Good	0 (0.0)	7 (38.9)	0.01
Other	16 (100.0)	11 (61.1)	
Periodontal status			
No periodontitis	3 (18.8)	5 (27.8)	0.42
Periodontitis	13 (81.3)	13 (72.2)	

CTCAE, Common Terminology Criteria for Adverse Events. * Fisher’s exact test.

## Data Availability

The data that support the findings of this study are available on request from the corresponding author, R.Y. The data are not publicly available due to restrictions, e.g., their containing information that could compromise the privacy of research participants.

## References

[1] Short M.W., Burgers K.G., Fry V.T. (2017). Esophageal Cancer. Am. Fam. Physician.

[2] Sjoquist K.M., Burmeister B.H., Smithers B.M., Zalcberg J.R., Simes R.J., Barbour A., Gebski V. (2011). Australasian Gastro-Intestinal Trials Group. Survival after neoadjuvant chemotherapy or chemoradiotherapy for resectable oesophageal carcinoma: An updated meta-analysis. Lancet Oncol..

[3] Huddy J.R., Huddy F.M.S., Markar S.R., Tucker O. (2018). Nutritional optimization during neoadjuvant therapy prior to surgical resection of esophageal cancer-a narrative review. Dis. Esophagus.

[4] Steenhagen E. (2019). Preoperative nutritional optimization of esophageal cancer patients. J. Thorac. Dis..

[5] Sakai M., Sohda M., Miyazaki T., Yoshida T., Kumakura Y., Honjo H., Hara K., Ozawa D., Suzuki S., Tanaka N. (2018). Association of Preoperative Nutritional Status with Prognosis in Patients with Esophageal Cancer Undergoing Salvage Esophagectomy. Anticancer. Res..

[6] Takagi K., Buettner S., Ijzermans J.N.M., Wijnhoven B.P.L. (2020). Systematic Review on the Controlling Nutritional Status (CONUT) Score in Patients Undergoing Esophagectomy for Esophageal Cancer. Anticancer Res..

[7] Onodera T., Goseki N., Kosaki G. (1984). Prognostic nutritional index in gastrointestinal surgery of malnourished cancer patients. Nihon Geka Gakkai Zasshi.

[8] Hikage M., Taniyama Y., Sakurai T., Sato C., Takaya K., Okamoto H., Konno T., Ujiie N., Naitoh T., Unno M. (2019). The Influence of the Perioperative Nutritional Status on the Survival Outcomes for Esophageal Cancer Patients with Neoadjuvant Chemotherapy. Ann. Surg. Oncol..

[9] Yamanaka-Kohno R., Shirakawa Y., Inoue-Minakuchi M., Yokoi A., Muro M., Kosaki H., Tanabe S., Fujiwara T., Morita M. (2021). Association of dental occlusal support with the Prognostic Nutritional Index in patients with esophageal cancer who underwent esophagectomy. Esophagus.

[10] Ueno M., Yanagisawa T., Shinada K., Ohara S., Kawaguchi Y. (2008). Masticatory ability and functional tooth units in Japanese adults. J. Oral Rehabil..

[11] Adiatman M., Ueno M., Ohnuki M., Hakuta C., Shinada K., Kawaguchi Y. (2013). Functional tooth units and nutritional status of older people in care homes in Indonesia. Gerodontology.

[12] Samnieng P., Ueno M., Shinada K., Zaitsu T., Wright F.A., Kawaguchi Y. (2011). Oral health status and chewing ability is related to mini-nutritional assessment results in an older adult population in Thailand. J. Nutr. Gerontol. Geriatr..

[13] Wu X., Shen J., Zhang X., Liu B., Liu M., Shi J., Qian S., Zong G., Lai H., Yuan C. (2024). The potential causal path between periodontitis stage diagnosis and vegetable consumption is mediated by loss of posterior functional tooth units and masticatory function. J. Clin. Periodontol..

[14] Teranishi R., Yamamoto K., Kurokawa Y., Uchihashi T., Sugauchi A., Tanikawa C., Kubo K., Takahashi T., Saito T., Momose K. (2023). Oral frailty is a risk factor for body weight loss after gastrectomy: A single-center, retrospective study. Int. J. Clin. Oncol..

[15] World Health Organization (1997). Oral Health Survey: Basic Methods.

[16] Ueno M., Yanagisawa T., Shinada K., Ohara S., Kawaguchi Y. (2010). Category of functional tooth units in relation to the number of teeth and masticatory ability in Japanese adults. Clin. Oral Investig..

[17] Henderson A.R. (2006). Testing experimental data for univariate normality. Clin. Chim. Acta..

[18] Bossi P., De Luca R., Ciani O., D’Angelo E., Caccialanza R. (2022). Malnutrition management in oncology: An expert view on controversial issues and future perspectives. Front. Oncol..

[19] Bossi P., Delrio P., Mascheroni A., Zanetti M. (2021). The spectrum of Malnutrition/Cachexia/Sarcopenia in oncology according to different cancer types and settings: A narrative review. Nutrients.

[20] Anandavadivelan P., Lagergren P. (2016). Cachexia in patients with esophageal cancer. Nat. Rev. Clin. Oncol..

[21] Muscaritoli M., Arends J., Bachmann P., Baracos V., Barthelemy N., Bertz H., Bozzetti F., Hütterer E., Isenring E., Kaasa S. (2021). ESPEN practical guideline: Clinical nutrition in cancer. Clin. Nutr..

[22] Shirakawa Y., Noma K., Maeda N., Tanabe S., Sakurama K., Sonoyama-Hanaoka A., Yoshitomi A., Kohno-Yamanaka R., Soga Y., Fujiwara T. (2021). Early intervention of the perioperative multidisciplinary team approach decreases the adverse events during neoadjuvant chemotherapy for esophageal cancer patients. Esophagus.

[23] Yamada S.I., Koike K., Isomura E.T., Chikazu D., Yamagata K., Iikubo M., Hino S., Hibi H., Katsura K., Nakamura S. (2021). The effects of perioperative oral management on perioperative serum albumin levels in patients treated surgically under general anesthesia: A multicenter retrospective analysis in Japan. Medicine.

[24] Takahashi-Arimasa K., Kohno-Yamanaka R., Soga Y., Miura R., Morita M. (2019). Efficacy of oral care provided by interprofessional collaboration for a patient with esophageal cancer associated with post-polio syndrome during neoadjuvant chemotherapy. Acta. Med. Okayama.

[25] Faul F., Erdfelder E., Lang A.G., Buchner A. (2007). G*Power 3: A flexible statistical power analysis program for the social, behavioral, and biomedical sciences. Behav. Res. Methods.

[26] Faul F., Erdfelder E., Buchner A., Lang A.G. (2009). Statistical power analyses using G*Power 3.1: Tests for correlation and regression analyses. Behav. Res. Methods.

